# Movement Sonification: Effects on Motor Learning beyond Rhythmic Adjustments

**DOI:** 10.3389/fnins.2016.00219

**Published:** 2016-05-27

**Authors:** Alfred O. Effenberg, Ursula Fehse, Gerd Schmitz, Bjoern Krueger, Heinz Mechling

**Affiliations:** ^1^Faculty of Humanities, Institute of Sports Science, Leibniz Universität HannoverHanover, Germany; ^2^Computer Science, Faculty of Mathematics and Natural Sciences, Institute of Computer Science II, University of BonnBonn, Germany; ^3^Institute of Sport Gerontology, German Sport University CologneCologne, Germany

**Keywords:** audiovisual information, motor learning, motor perception, motor rehabilitation, movement sonification, multisensory integration

## Abstract

Motor learning is based on motor perception and emergent perceptual-motor representations. A lot of behavioral research is related to single perceptual modalities but during last two decades the contribution of multimodal perception on motor behavior was discovered more and more. A growing number of studies indicates an enhanced impact of multimodal stimuli on motor perception, motor control and motor learning in terms of better precision and higher reliability of the related actions. Behavioral research is supported by neurophysiological data, revealing that multisensory integration supports motor control and learning. But the overwhelming part of both research lines is dedicated to basic research. Besides research in the domains of music, dance and motor rehabilitation, there is almost no evidence for enhanced effectiveness of multisensory information on learning of gross motor skills. To reduce this gap, movement sonification is used here in applied research on motor learning in sports. Based on the current knowledge on the multimodal organization of the perceptual system, we generate additional real-time movement information being suitable for integration with perceptual feedback streams of visual and proprioceptive modality. With ongoing training, synchronously processed auditory information should be initially integrated into the emerging internal models, enhancing the efficacy of motor learning. This is achieved by a direct mapping of kinematic and dynamic motion parameters to electronic sounds, resulting in continuous auditory and convergent audiovisual or audio-proprioceptive stimulus arrays. In sharp contrast to other approaches using acoustic information as error-feedback in motor learning settings, we try to generate additional movement information suitable for acceleration and enhancement of adequate sensorimotor representations and processible below the level of consciousness. In the experimental setting, participants were asked to learn a closed motor skill (technique acquisition of indoor rowing). One group was treated with visual information and two groups with audiovisual information (sonification vs. natural sounds). For all three groups learning became evident and remained stable. Participants treated with additional movement sonification showed better performance compared to both other groups. Results indicate that movement sonification enhances motor learning of a complex gross motor skill—even exceeding usually expected acoustic rhythmic effects on motor learning.

## Introduction

When looking back to our sport classes, recalling how breaststroke swimming, the overhand technique in volleyball or even rowing was taught, we remember our teachers explaining and demonstrating the techniques. Technique acquisition in sports is usually shaped by visual demonstrations and verbal information as getting evident in popular sportscientific textbooks (Newell and Corcos, 1993; Schmidt and Lee, 2005). Also in perceptually directed research in sport science, processes of motor perception, motor control and motor learning have been studied primarily related to single sensory modalities and dominated by the visual domain (Williams et al., 1999, 2004; Abernethy, 2013). But on a closer view, motor behavior is a multimodal phenomenon: Motion can not only be observed visually but also perceived by the auditory and the tactile sense, and perception of one's own motion is just as well based on visual, auditory, kinesthetic, vestibular, and tactile information. Recent behavioral—as well as neurophysiological—research therefore focusses increasingly on audiomotor and multisensory contributions to the regulation of behavior (Frassinetti et al., 2002; Soto-Faraco et al., 2003; Calvert et al., 2004). Even though majority of work is localized in the field of basic research, also applied studies address the area of complex gross-motor behavior, often with a close link to biological motion perception (Barraclough et al., 2005; Bidet-Caulet et al., 2005; Mendonca et al., 2011).

Up to now, only a few studies are dealing with the multisensory influence on motor learning, and this is especially given for applied research related to gross motor motion, as being typical for sports. Therefore, the introduction will focus firstly on audiomotor information processing to identify the perceptual characteristics of audition, getting effective besides visual information on the regulation of behavior (Haueisen and Knoesche, 2001; Bangert and Altenmüller, 2003; Haslinger et al., 2005; Lahav et al., 2007). Afterwards multisensory perception is taken into account, with the focus on mechanisms of audiovisual information processing and related behavioral benefits. Then some studies using sonification to support motor control and motor learning are introduced.

Findings on the emergence of audiomotor co-activations and the multisensory integration mechanisms will be taken in consideration to determine how additional movement acoustics could be shaped to address audiomotor functions as well as multisensory integration sites within the central nervous system (CNS). With other words: How could an effective movement sonification be tailored and how could it get effective on motor learning? Here some neurophysiological work will be consulted. Based on these findings the own method of movement sonification will be developed combining dynamic and kinematic movement parameters into a 4-dimensional sonification. Movement sonification was applied in the present study to support motor learning in technique acquisition of indoor-rowing. To evaluate the impact of an additional movement sonification three groups were treated with different kinds of instructions and feedback over a training period of 3 weeks: One group was treated with visual information (video instruction + concurrent video feedback) and two groups with different kinds of audiovisual information (video/sonification instruction & real-time video/sonification feedback; video/motion attendant sound instruction & real-time video/motion attendant sounds feedback).

### Music making—acoustic information of motor behavior generated by the auditory system

In a first step, music related research will be focused. In the music domain research on motor learning is related to auditory, especially musical perception and thus is an appreciated supplement to visually dominated motor learning research in the fields of sport, ergonomics or motor rehabilitation. More than this: Music making is a domain of motor behavior with excellent subtle and unambiguous feedback about the precision and the quality of motor control. On music making, quality of motor control can be assessed immediately via the acoustical or musical result resp. When for example playing the keyboard, the spatial accuracy of actions can be assessed via the sound frequency or tone pitch sequence, the dynamical precision via the sound amplitude and the temporal exactness via the duration of sounds and pauses as well as via constancy of metrum and shape of rhythm. With other words: Auditory and especially musical perceptual skills are very well-suitable for analyzing several important qualities of motor control. But what are the appropriate perceptual and motor features established into the brain of an expert musician? The work of Haueisen and Knoesche (2001) indicates that on expert pianists, when listening to a piece of one-handed piano music, motor areas primarily related to the analogous effector get co-activated.

Obviously, specific audiomotor networks get established in music experts—at least after years of exercise. But how long does it take till such sensorimotor networks get initially established? There already exists some empirical evidence that even on novices it does not take long time till auditory-motor co-activations appear within the human brain: Bangert and Altenmüller (2003) reported a fast emergence of audiomotor co-activation patterns on musical novices learning to play a simple melody on a keyboard. In an EEG-study, the authors reported audiomotor co-activations which develop within short temporal intervals of about 20 min of practice and get firmly established within a few weeks of exercise—nevertheless with a specific shape. Only the replay of the before trained simple melody led to motor co-activation, whereas new melodies, played on the same keyboard, did not.

### Non-musical acoustic information on motor behavior

When dealing with acoustic information related to motor behavior, also natural motion attendant sound and the information about the related action as well as about the related actor have to be taken into account. During last years, a growing body of empirical studies on the information coded within natural motion sounds has been acquired. Early evidence about the impact of motion attendant sounds on motor performance has been presented by Takeuchi (1993) on tennis players indicating that auditory perception of tennis ball sounds (stroke and landing) supports a high performance and deprivation of auditory perception reduces the performance. The information coded within natural motion sounds was described by Effenberg (1996) and used as an initial point for the development of an ecological framework of movement sonification. In 2004, Agostini et al. demonstrated that athletes were able to use auditory models of hammer throws to improve their performance. The high amount of information mediated by natural movement sounds has also been illustrated by more recent studies using the own/other paradigm (Murgia et al., 2012; Kennel et al., 2014). Beyond that basic physiological parameters like the breath duration can be affected subconsciously by listening to the ecological sound of breathing even more compared to artificial sound and thereby indicating a demanding influence of natural sounds (Murgia et al., 2016). On the other hand complex artificial movement sound is powerful to allow subtle distinctions of own vs. other movement patterns as shown related to movement sonification of indoor rowing by Schmitz and Effenberg (2012). Meanwhile there exists numerous evidence about acoustically coded information in natural movement sounds and about the subtle impact on motor behavior (Sors et al., 2015). Supportive fMRI research has been published by Woods et al. (2014) e.g., indicating, that expertise in a certain sport is an important factor related to the way sport specific acoustic information is processed in the brain: Experts, familiar with the presented sport specific sounds showed greater neural activation in sensorimotor areas and areas responsible for auditory and motor planning (Agostini et al., 2004).

### Music making and audiovisual information processing

Even though neurophysiological research on motor learning in the area of music is dominated by audiomotor relations, also audiovisual-motor interactions have been focused. Haslinger et al. (2005) found in an fMRI-study that pure observation of piano playing recruited auditory areas in experienced pianists and discussed the participation of mirror neurons within the inferior fronto-parieto-temporal network. Taken together with the findings of Bangert and Altenmüller (2003), it is getting obviously that learning to play an instrument results in relatively fixed co-activations between different perceptual—at least auditory and visual—and motor networks. If once established, visual stimulation is sufficient for co-activation of auditory areas as well as for co-activation of motor-related networks. Also auditory simulation leads to co-activation of motor-related networks. These findings got further support with an fMRI-study from Lahav et al. (2007) showing that such audiomotor as well as audiovisuomotor co-activations are case specific, that a certain co-activation pattern is referring to a certain musical pattern resp.: The established co-activation of motor subsystems only became evident if the melody, learned to play before, was hearable. Activation of the motor network was much smaller when the order of the notes was changed and it disappeared by “motorically” unknown—untrained—melodies. The findings of Lahav et al. indicate that there is a common hearing-doing system, that is highly dependent on the individual's motor repertoire, is getting established rapidly and is likely not limited to the field of music (Lahav et al., 2007).

### Non-musical audiovisual information processing

For some years not only studies on audiomotor functions have been growing in number (Bangert et al., 2006), also crossmodal interactions related to the perceptual system as well as to motor control and motor learning are getting more and more into the focus of research (Seitz et al., 2006). The quality of perception is usually enhanced if distal events are perceived by at least two different senses compared to unimodal perception. Numerous studies on multisensory stimuli effectiveness on different aspects of behavior have been realized meanwhile and most of them deliver supporting evidence as realized by Vroomen and de Gelder (2000) showing with a stimulus detection task in a rapidly changing sequence of visual distractors. Further perceptual effects of audiovisual convergent stimuli have been described by Frassinetti et al. (2002) in terms of an increased detection rate of low intensity stimuli. Also Seitz et al. (2006) reported an enhanced ability for the detection and discrimination of coherent motion pattern for audiovisual trained subjects compared to unimodal trained ones. The discrimination ability of audiovisually presented objects was studied by Giard and Peronnet (1999), who reported more reliable discriminations for audiovisually coded transforming objects compared to unimodal presentation. Those enhanced activation correlates with better performance in detecting coherent motion.

Further studies in the field of applied research indicated enhanced effectiveness of convergent audiovisual information on motor perception and even on motor perception and motor control: Evidence on motor performance was delivered by Chiari et al. (2005) in terms of a real-time audio feedback on trunk kinematics enhancing the control of body sway. Rath and Rocchesso (2005) demonstrated that a tilting bar can be handled with a higher precision if additional motion acoustics are available. Furthermore, gross-motor sport movements can be assessed and reproduced with higher precision under an audiovisual condition compared to pure visual treatment as reported by Effenberg (2005).

Also on motor learning there exist some evidence on the supportive function of additional auditory information: Shea et al. (2001) have revealed supporting effectiveness of an additional auditory model. The authors observed besides a better performance of a simple motion pattern (rhythmic finger movement) even a more effective learning process: Required time was reduced and precision in terms of absolute as well as relative timing was enhanced. Later work of Kennedy et al. (2013) confirmed an enhanced temporal stability (retention) of a before learned 2:3 bimanual tapping task especially for an audiovisual model. More recently Danna et al. (2013, 2015) reported first indications that also less rhythmic finemotor learning (handwriting acquisition of children) can be supported by additional auditory cues. Similar results had been presented recently by Effenberg et al. (2015): The acquisition of character handwriting of young children had been supported by an additional real-time sonification of the writing trace (“SoundScript”) in terms of an accerleration of the emergence of model-like character patterns. And the learning of gross motor movements—as they are typical on sports—has been supported with concurrent auditory feedback by Baudry et al. (2006). The authors found a time-stable benefit for the body segmental alignment on a circle movement performed on a pommel horse based on a training with concurrent auditory feedback. And most recent work from our workgroup delivered first evidence on the effectiveness of a 4-dimensional kinematic movement sonification in stroke rehabilitation on hemiparesis of the upper limbs after a 5-day training of everyday actions about only 20 min daily (Schmitz et al., 2014).

### Neurophysiological background

In addition to the behavioral findings primarily neurophysiologically intended research has been conducted in the last years focussing on the underlying mechanisms of multisensory integration responsible for performance enhancement. Cortical as well as subcortical multisensory integration sites are addressed by convergent multisensory stimuli in addition to unimodal visual and auditory functions and may be jointly responsible for perceptual and behavioral benefits (Calvert and Thesen, 2004; Beauchamp, 2005). Dedicated to gross-motor human motion perception there exists evidence on integrating motion sound within an area responsible for ‘visual’ biological motion perception, the posterior superior temporal sulcus (STSp; Bidet-Caulet et al., 2005). The first fMRI-study dedicated to the exploration of multisensory integration based on movement sonification was realized by our workgroup: Scheef et al. (2009) revealing, that an audiovisual stimulus of a counter-movement-jump (video and sonification of the ground reaction force) evokes real multisensory integration effects in terms of a supra-additive activation enhancement in Area V5/MT as well as enhanced activation in STS bilaterally, which both playing a role in audiovisual perception of biological motion.

Even if there exists only some evidence for direct connections between audiovisual perception and motor execution on humans—as described for the superior colliculus on cats by Stein and Meredith (1993) and Rowland and Stein (2008), the existence of audiovisual mirror neurons in the monkey brain has been demonstrated with single cell recordings realized by Kohler et al. (2002) and is also discussed on humans (Keysers et al., 2003). Baumann and Greenlee (2006) showed via fMRI on humans that convergent audiovisual motion stimuli evoke substantially larger activation in different brain areas (cluster within the superior temporal gyrus, supramarginal gyrus, superior parietal lobule, and cerebellum) compared to the addition of both unimodal activations.

Resent research from our workgroup (Schmitz et al., 2013) is indicating, that the activation of the mirror neuron system increases when convergent audiovisual information in terms of a movement sonification of a moving avatar is available to the perceptual systems. The superior temporal sulcus, inferior parietal cortex and premotor regions as well as subcortical structures showed enhanced activation of the action-observation-system in comparison to similar, but divergent audiovisual stimuli. Beyond this also key-players of the striato-thalamo-frontal motor loop got increasingly activated—which had been observed on untrained participants with no or nearly no experience in movement sonification. Though it has not been a new finding that short-term plasticity related to motor learning can appear even within minutes, it is important for our issue that based on artifical movement sound sensorimotor co-activation in terms of audiomotor or even audiovisual-motor co-activation emerges also on “novices” instantly. The term “novices” refers to participants without experience with movement sonification, but all of them were able to breaststroke. Taken together this is some functional evidence that enhancing motor execution with artifical movement acoustics is efficient to address additionally audiomotor as well as audiovisual-motor mechanisms within the context of motor learning.

### Interim resume

Music making and listening indicates the enormous capacity of the auditory system to generate and mediate information about movements. The observed prompt emergence of case specific audiomotor co-activation patterns indicated the rapid dynamics of neuronal plasticity with a high sensitivity for the specific shape of the referenced movement-acoustic—time-varying—stimuli. Also on non-musical behavior basal motion-related perceptual functions benefit from multisensory information processing as well as processes of perception and motor control of human movements. And even beyond that—motor learning is getting more effective and stable if based on multisensory information, as shown for fine-motor, rhythmic hand-/finger-movements and also for more complex writing-movements and even in first steps for a certain feature of a gross-motor sport-movement on a pommel horse as well as in stroke rehabilitation of hemiparesis. A sharp plea for multisensory training is given by Shams and Seitz (2008) referring to the additional integration of intermodal processing and multimodal integration functions in learning. Such findings are supported by a statistical approach from Ernst and Bülthoff (2004) explaining the emergence of additional information from multisensory integration. And finally some more recent research from Shams et al. (2011) should be mentioned here indicating furthermore a retroaction of multisensory—audiovisual—training to the perception within each single modality.

### Creating the method of real-time movement sonification

The method of movement sonification is used by a growing number of researchers, when combining movement data with sound. We use this term, which we have created first in 2005 (Effenberg, 2005) related to motor perception and motor control, for the acoustic transformation of kinematic and/or dynamic movement data. The fundamental idea is to tune in the ear into the process of motor perception, related to external motion of others (trainers, functioning as “models”) as well as to one's own movements when functioning as an additional feedback channel to support, to enhance and to shape the emergent sensorimotor representations in terms of “internal models” (Wolpert et al., 1995, 2011). Considering that Sigrist et al. (2015) failed to generate a long-lasting learning benefit based on sonified acoustic error information on indoor rowing, which was consisting of only one dimension of one external movement feature during one phase of the movement cycle (the horizontal angle of the rowing oar during the recovery phase), we generate a continuous 4-dimensional movement acoustics based on two kinematic and two dynamic movement parameters.

This kind of real-time movement sonification is configured to be used for audiomotor processing as well as for enhancing and shaping multisensory representations efficiently. In contrast to the mode of “error-feedback,” as considered by Sigrist et al. (2013), the processing of our kind of movement acoustics is not dependent on conscious cognitive processing, because the processing—even multisensory integration—is mandatory if the stimulus is hearable and certain criteria of intermodal convergence are fulfilled. Resulting in an enhanced spectrum of movement information, usable as instruction as well as feedback, this kind of information supports the emergence of adequate sensorimotor/perceptuomotor representations (internal models). The used method is described in more detail in the following section. Here we conclude the first section with three research hypotheses:

H1: Participants of both groups treated with convergent audiovisual information show better learning results in terms of a steeper learning curve/a faster approximation to the model technique.
H2: Audiovisually treated groups are more precise in rhythmic demands of the indoor rowing technique.
H3: Participants treated with complex sonification benefit in terms of a better coordination of the movement resulting in higher technical performance.

## Methods

Novices were asked to learn the basic technique of indoor rowing by visual or audiovisual instruction and feedback. When instructing the participants it was pointed out that the primary aim was not a maximum intensity of indoor rowing, but to reproduce the technical pattern as precisely as possible. The quality of the technical pattern was operationalized by four movement parameters: grip force and footrest forces as dynamic parameters and grip pull-out length and sliding seat position as kinematic parameters. The participants' courses of these parameters during pull-out phase were compared to the model's ones.

The similarity of the participants' and the model's technique was computed by using a DTW-algorithm designed for similarity calculation of two temporal sequences differing in length. Such DTW-algorithms have already been used in different contexts like speech recognition and analysis of motion capture data as described in Rabiner and Juang (1993), Forbes and Fiume (2005) and Demuth et al. (2006).

### Experimental design

**Figure 1:**
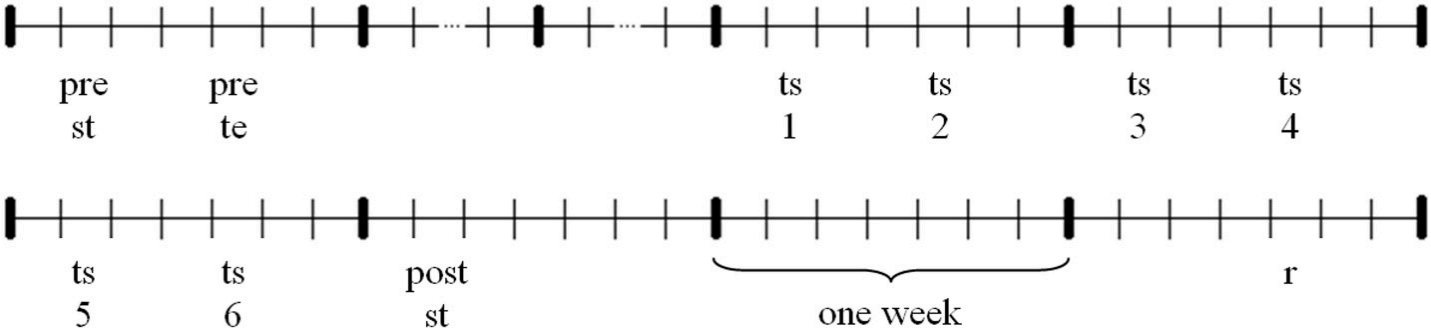
**Experimental procedure: pre st, strength pretest; pre te, technique pretest; ts1-6, training session 1–6; post st, strength posttest; r, technique retention test**.

The experimental procedure extended to about 9 weeks, whereof the training period took 3 weeks. Participants were asked to acquire a basic technique of indoor rowing, demonstrated by a professional rowing athlete. The experimental procedure is visualized in Figure 1. With the initial two pretests data about the initial individual technique level as well as strength data were collected. Afterwards the total sample was divided up into three subsamples, parallelized on initial technique level and age. All three samples completed the same 3-week training period each obtaining different kinds of information in terms of instruction and real-time feedback. Participants trained two times a week. The procedure of a single training session is explained below. One week after the last training session a strength posttest was conducted. Three weeks after the last training session participants completed a technique retention test finally.

### Participants

48 male volunteers participated in the experiment, all of them without any experience in rowing (mean age = 22.8 ± 5.0). All participants showed normal or corrected-to-normal vision and normal hearing^1^. Though the Central Ethics Commission (CEC) at Leibniz University Hannover (LUH) was starting the assessment service for the first time not before 2012, the beginning of subject recruitment was started without a specific ethical approval but in accordance with the Ethics Guidelines of the “German Psychological Society” also including informed consent declaration, privacy and confidentiality and the final presentation of research results to all participants. All participants gave their written consent to participate in this psychological-behavioral study.

### Experimental conditions

All three subsamples ran through the same training procedure, each with a different kind of information in terms of instruction and real-time feedback.

- Visual condition (V): Treatment group V was only treated with video information.
- Natural audiovisual condition (AV_nat_): Treatment group AV_nat_ was treated with video and natural motion attendant sounds.
- Sonified audiovisual condition (AV_soni_): Treatment group AV_soni_ was treated with video and movement sonification.

### Stimulus material

#### Visual stimuli

**Figure 2:**
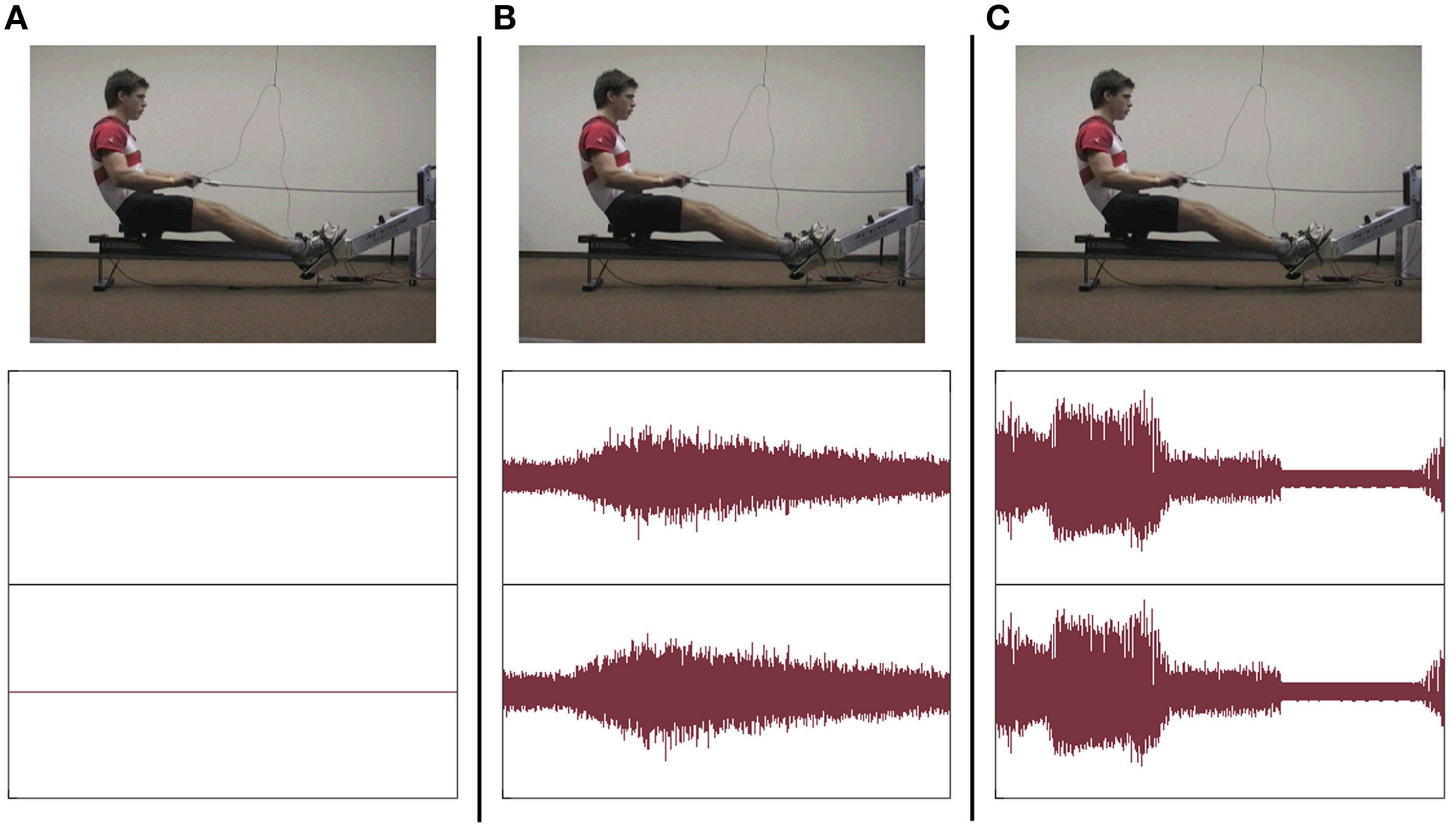
**Visual and auditory stimuli under the three conditions: frame of instruction video (above) + sound pressure level of soundtrack of a single rowing cycle (below); (A) group V, (B) group AV_*nat*_, and (C) group AV_*soni*_**.

Instruction videos (rowing model) and feedback videos (participants' performance) were taken from a lateral view (see Figure 2). Videos were projected on a big screen (260 × 195 cm) in front of the rowing ergometer type Concept II. For instruction and feedback, videos were presented with Sony Video Capture 6.0b.

#### Auditory stimuli

Both kinds of auditory stimuli were presented via headphones. For group AV_nat_ the sound of the rowing ergometer flywheel and the sliding seat were taped with a directional microphone^2^ and mediated via headphones^3^. For group AV_soni_ movement sonification based on two kinematic and two dynamic motion data streams that were transformed in real-time to multichannel continuous motion-sound. Data streams of four different sensors were acoustically represented: grip force, sum of footrest forces, grip pull-out length, and sliding seat position. In Figure 2 visual and auditory stimuli under the three experimental conditions are depicted.

**Figure 3:**
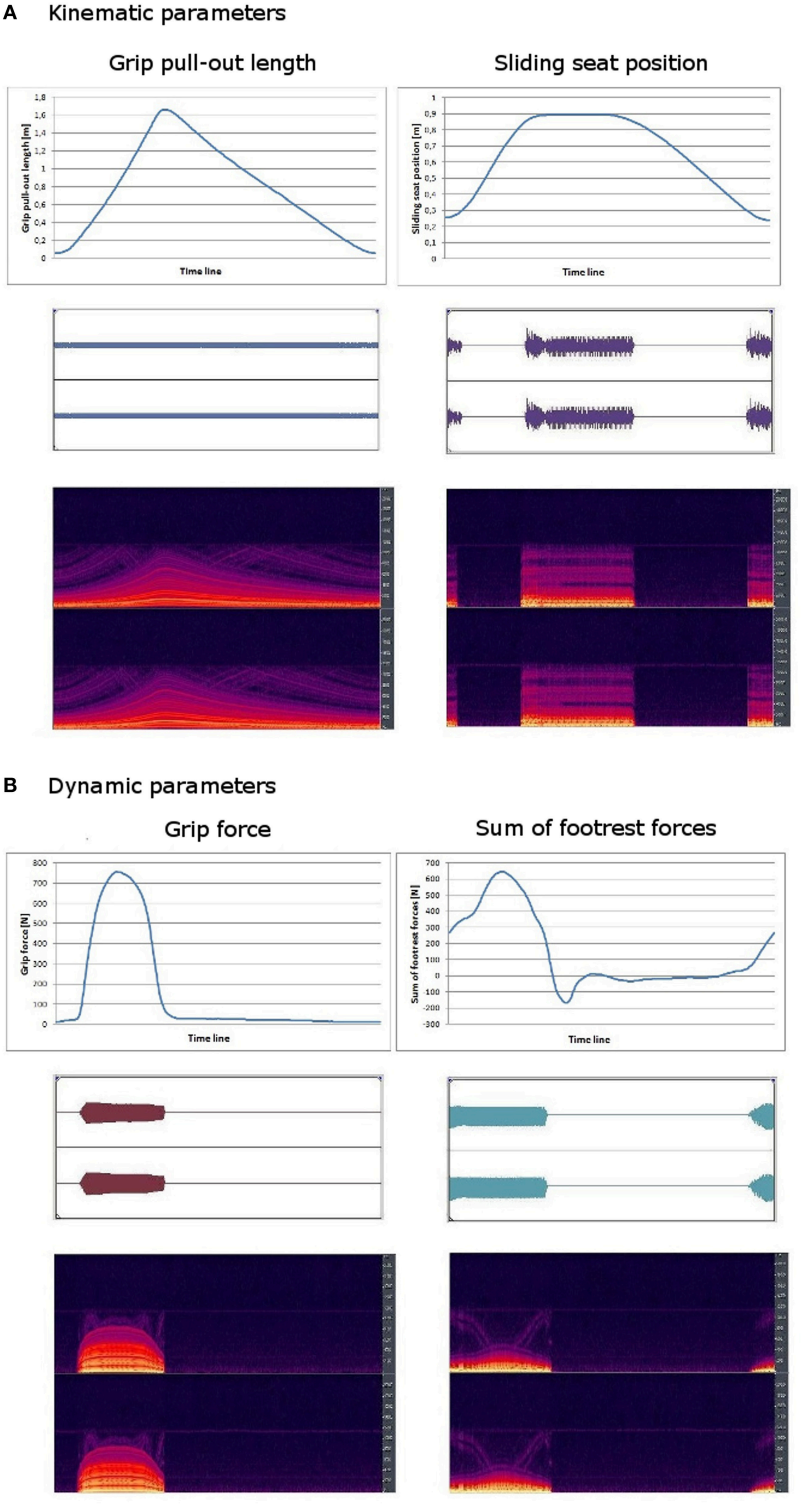
**(A)** Kinematic parameters; **(B)** Dynamic parameters. Data curve (up), amplitude level diagram (middle) and spectral graph (down) of the four data channels of a single rowing cycle (3.03 s).

Kinematic and dynamic data were recorded using FES-Software^4^ and transmitted to Lab-View-Software^5^ and further on to sonification-software^6^. The sonification-software received data of grip force, footrest forces, grip pull-out length and sliding seat position. Movement data were systematically mapped on sound features: each data stream was used to modulate frequency and partially also amplitude of a midi sound. Grip pull-out, grip force and footrest forces were represented continuously. For both force parameters a muting level was defined for values near around zero as well as for negative values. So forces could only be acoustically perceived when they were also kinesthetically clearly perceivable. By using a muting level oscillating sounds for fast changing forces near around zero were avoided. In contrast to the three continuously transformed parameters, sliding seat position was sonified event-related: it could be only heard at maximum and minimum position. Independently of effectively exerted force and realized grip pull-out length and sliding seat position, the frequency interval was chosen in a manner that maximum and minimum of a single data stream was related to the same frequency for each individual in each training session. Sonification of rowing model was produced the same way. This kind of normalization enables participants to produce the same sound pattern as the model, independently of individual absolute strength abilities and individual anthropometry. Figure 3 shows the data curves of the kinematic **(A)** and the dynamic **(B)** parameters and the characteristics of the resulting sounds.

#### Instruction

For the instruction-video the motion pattern of Eric Johannesen^7^ performing on the Concept II rowing ergometer (same rowing ergometer as used in the study, but higher drag factor) was videotaped. The videosequence contained 10 cycles of rowing, each lasting 3 s. Pull-out phase and recovery phase had a time ratio of 2:1. Depending on the treatment, there was no motion attendant soundtrack (V), the soundtrack contained natural motion attendant sounds of the rowing ergometer (AV_nat_) or the model's movement sonification (AV_soni_) resp.

#### Feedback

As feedback, participants observed their own rowing in real-time for ten cycles in the middle of each training block of 50 cycles. Depending on the treatment they heard no motion attendant sounds, their natural motion attendant sounds or their own movement sonification in real-time.

**Figure 4:**
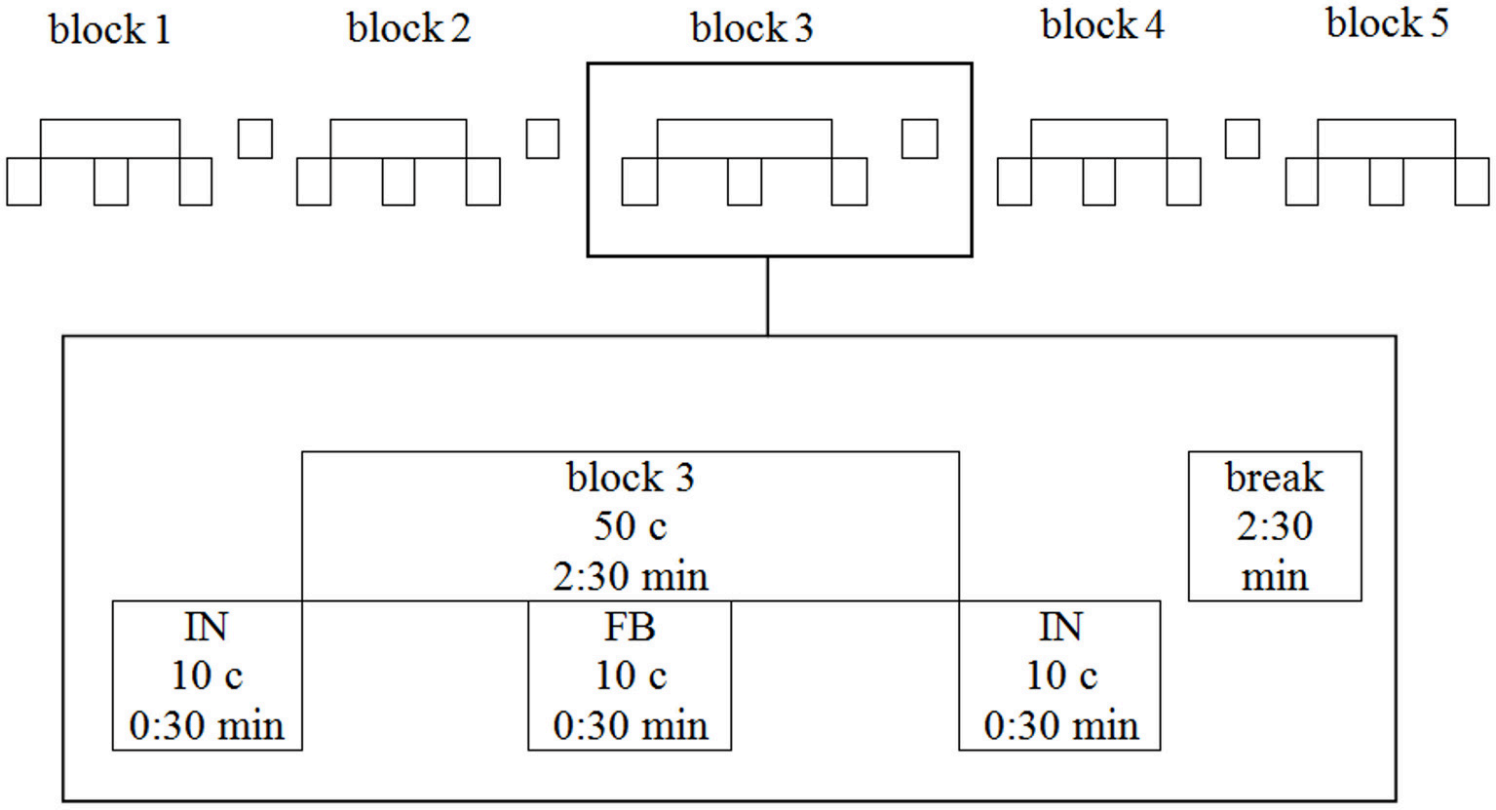
**The five training blocks of a single training session**. One block is depicted magnified. IN, instruction; FB, feedback; c, cycles.

To mask natural motion attendant sounds all participants heard noise (sea rushing) via headphones while there was no auditory instruction or feedback. The chronology of sea rushing audio and audio-feedback within each block of training is illustrated in Figure 4: Audiovisual treatment groups heard 20 cycles of sea rushing followed by 10 feedback cycles (motion attendant sound or movement sonification resp.) followed by another 20 cycles of sea rushing.

### Procedure

#### Pretest

After warming up, making themselves familiar with the rowing ergometer and watching the rowing model for ten cycles participants completed the technique pretest by rowing for 30 cycles without any feedback.

#### Training

One training session consisted of five training-blocks, each lasting for 2.5 min followed by 2.5 min break afterwards (see Figure 4). After the presentation of the model's technique as instruction, the participants started to row for 50 cycles. From cycles 21 to 30 the participants' own technique was presented as real-time feedback. Following the 50th cycle, the presentation of the instruction video was repeated.

Participants were instructed to align their rowing technique to the model's technique. In addition, they were told to tune their cycle frequency to 20 cycles per minute by watching the frequency display. To assure that participants do not tire—which would cause interferences with the quality of technique—they were instructed to row with comfortable effort. Group AV_soni_ was not informed in detail how sound was composed, they only knew that it was configured by their motion.

#### Retention

In retention participants completed one block of rowing without any instruction or feedback. Due to the long break between the last training session and retention of about 3 weeks, a warm-up of 50 cycles was realized before the retention block.

### Data acquisition and data analysis

Personal data (name, sex, date of birth, health data etc.) were collected by questionnaire and made anonymous afterwards. In strength pretest and strength posttest isometric maximum strength of leg extension and arm flexion were measured with a leg-press (knee-angle: 90°) and a row machine (anteversion shoulder: 30°). Participants conducted the tests after warming up and making themselves familiar with the equipment. To determine the individual maximum strength, the best out of three trials was used. Because strength abilities generate an impact on motor performance, the development of strength was controlled by comparing data of strength pre- and posttests.

Four sensor systems were applied on the rowing ergometer: a resistance strain gauge for grip force (GF), two sensors for footrest forces (FF), and two incremental encoders each for grip pull-out length (GP) and sliding seat position (SS). All four parameters were recorded with 100 Hz, for footrest forces the sum of the two sensor streams was computed. For data analysis cycles 31–40 (21–30 for pretest) of each training block were selected. An average cycle was computed for each of the four raw data streams. In a second step data were normalized to eliminate differences in body size and individual strength. Grip pull-out and sliding seat data was normalized on values between 0 and 1, grip force and footrest forces data were only divided by the particular maximal value to maintain algebraic sign of measured values.

Using the dynamic-time-warping (DTW) algorithm (Müller, 2007) we calculated the distance values between the model's technique and participants' individual technique (normalized average pull-out phase) for each of the four parameters.

The corresponding procedures were performed for each of the regarded time series (normalized averaged curve of grip pull-out length, grip force, sum of footrest forces, sliding seat position) separately. As a result of the DTW algorithm we obtained an optimal alignment of the compared time series, a so called warping path. This warping path gives information how the time series have to be stretched or compressed to get an optimal matching. These temporal deformations do not have to be linear: some segments of a signal might be stretched while others are compressed to get the optimal alignment. We defined the accumulated costs along the warping path to be a distance measure to finally compare the time series.

The computation of an alignment using DTW can be divided into three basic steps:

1. Computation of local distance matrix (LDM).
2. Computation of a global distance matrix (GDM).
3. Search the GDM for the warping path.

These steps are now described in detail:

1. Computation of the LDM

The local distance matrix is an *m* × *n* matrix, where m is the number of data points of the model's technique curve (s) and n is the number of data points of the participant's individual technique curve (t). Each entry of the matrix corresponds to the absolute distance between one data point of the participant's technique curve and one data point of the model's technique curve. For example, the entry in the i-th row and the j-th column corresponds to the absolute distance between the i-th sample point of the participant's technique curve, and the j-th sample point of the model's technique curve.


for i: = 1 to n
       for j: = 1 to m
           LDM[i, j]: =  d(s[i], t[j])
 


2. Computation of the GDM

Based on the LDM we are now able to compute the GDM. The GDM is an *m*×*n* matrix, too. It represents the accumulated or global costs between the time series regarded. Here the entry in the i-th row and j-th column is the minimal cost for an optimal alignment if the time series would end at these frames. The GDM is computed by accumulating local distances stored in the LDM according to a special scheme. The value of the entry in the i-th row an j-th column is computed as:


GDM[i,j]: =  LDM[i,j] + minimum(GDM[i - 1,j],
      GDM[i,j - 1], GDM[i - 1,j - 1])
 


The computation starts at the entry [1, 1].


for i:= 1 to n
      for j:= 1 to m
          GDM[i, j]: =  LDM[i, j]
                     + minimum(GDM [i-1,j],
                               GDM [i,j-1],
                               GDM [i-1,j-1])
 


The entry [m, n] is taken as “vertical distance value.”

3. Search the warping path

After the computation of the GDM, we can now extract the optimal alignment. It leads from the entry [m, n] to the entry [1, 1], involving only single steps to the left, up or diagonally left up, leading to the entry with the lowest value, resp. The number of needed steps builds the “path length” and the ratio of “path length” and the minimal possible path length (in our case almost always m-1) builds the “horizontal distance value.” From vertical and horizontal distance value, a “distance value” was computed for each data stream. In order to respect the different dimensions of the two computed distance values, we decided to utilize Pythagorean theorem:

$$\begin{array}{l} \text{distance value}=\;\\ \sqrt{{\left(\text{vertical distance value}\right)}^{2}+\;{\left(\text{horizontal distance value}\right)}^{2}} \end{array}$$

To consider the rate of force, additionally a force index (FI) was built in terms of the average grip force during pull-out phase divided by the maximum strength value (MSV). Maximum strength value consisted of the mean of maximum strength data (sum of legpress & row machine) in strength pre- and posttest.

$$\begin{array}{l} \text{MSV}=((\text{maximum strength from legpress pretest}+\\ \text{ row machine pretest})\;+\;(\text{maximum strength from}\\ \text{ legpress posttest }+\text{row machine posttest}))/2\\ \text{ FI }=\text{average grip force during pull}-\text{out phase}/\text{MSV} \end{array}$$

**Figure 5:**
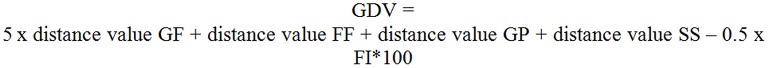
**Formula to compute the general distance value (GDV)**. GF, grip force; FF, footrest forces; GP, grip pull-out; SS, sliding seat; FI, force index. Distance value of the grip force is weighted five-fold, other distance values one-way, half of force index is subtracted.

A combination of the four distances values with the force index allowed to build a general distance value (GDV, see Figure 5) for each block (five blocks a training session multiplied by six training sessions + pretest, retention test). GF was weighted fivefold due to its dominant importance on the acceleration of a rowing boat. To take into account the two technique influencing aspects, a formula was developed fusing a coordinative dimension with a fitness dimension.

To consider the influence of the subcomponents of the GDV (grip force, footrest forces, grip pull-out, sliding seat, and force index) which had not been normalized before training, for each component data of each of the three treatment groups was normalized to the group mean for pretest. Additionally, a variability coefficient was computed by dividing the standard deviation of mean energy expended during grip pull-out by its mean, to quantify the stability of motion. Also duration of pull-out phase was computed to check the difference of the model's (1 s) and the participants' duration of pull-out phase.

## Results

### Learning effects

**Figure 6:**
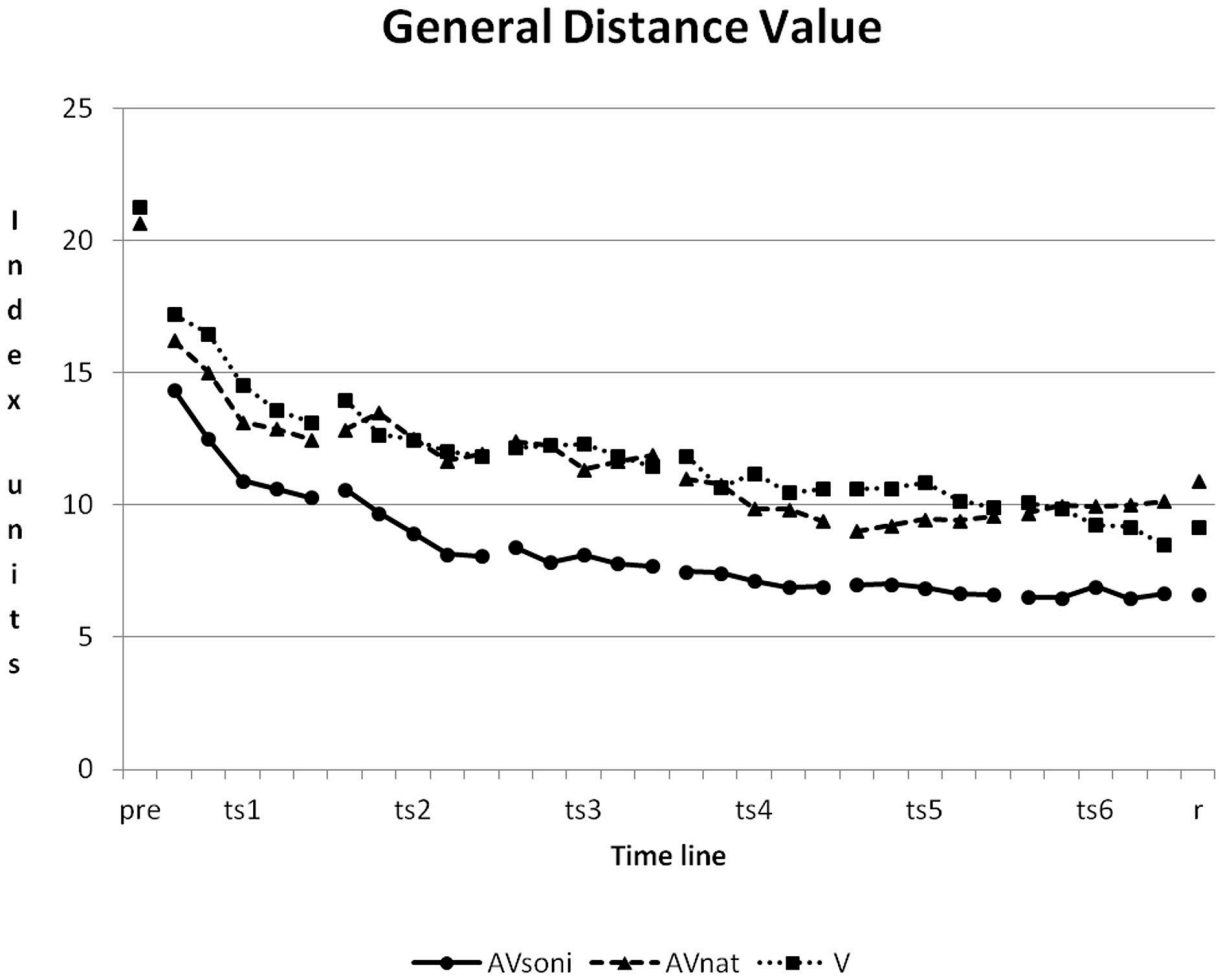
**Learning curves, showing the development of group means of GDV from pretest to retention test for all three treatment groups**. Pre, pretest; ts, training session; r, retention test; AV_soni_, treatment group AV_soni_; AV_nat_, treatment group AV_nat_; V, treatment group V. Standard deviations are regarded subsequently.

At the beginning of the study, a high variability of participants' rowing techniques was measurable. The average GDV of all participants was at 20.08 ± 10.46 (V: 21.29 ± 9.78, AV_nat_: 20.69 ± 10.31, AV_soni_: 21.27 ± 11.30) in pretest. In course of the study, participants approximated their rowing technique to the model's technique. The mean GDV of the last training session was 8.65 ± 3.99 (V: 9.38 ± 5.48, AV_nat_: 9.96 ± 4.45, AV_soni_: 6.62 ± 2.05). The learning curves of the three treatment groups are depicted in Figure 6.

A first strong reduction of GDV could be observed from the pretest to the first training session for all participants. The GDV in pretest was compared to the GDV at the first block in the first training session with an ANOVA r.m. revealing a significant main effect “time” [*F*_(1, 45)_ = 16.086, *p* < 0.001, $\eta_{\text{p}}^{2}=$ 0.26]. Neither main effect “treatment” [*F*_(2, 45)_ = 0.145, *p* = 0.865, $\eta_{\text{p}}^{2}=$ 0.01] nor interaction “time” × “treatment” [*F*_(2, 45)_ = 0.479, *p* = 0.623, $\eta_{\text{p}}^{2}=$ 0.02] became significant.

For the whole training ANOVA r.m. (6^*^5 data points) on GDV revealed significant main effects on “training session” (“ts,” six sessions in 3 weeks) [*F*_(5, 225)_ = 33.111, *p* < 0.001, $\eta_{\text{p}}^{2}=$ 0.42] and on “block” (“b,” five blocks per training session) [*F*_(4, 180)_ = 21.151, *p* < 0.001, $\eta_{\text{p}}^{2}=$ 0.32].

**Figure 7:**
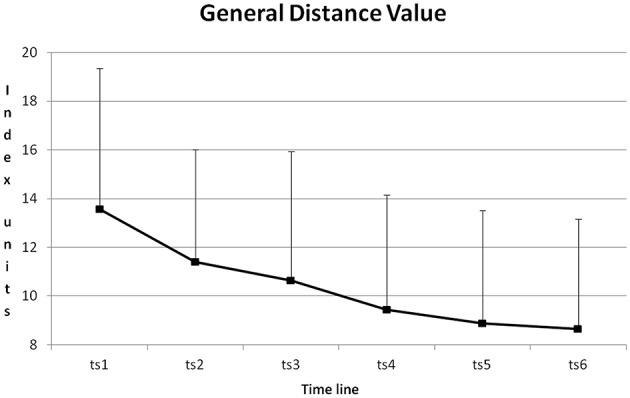
**Course of GDV and its standard deviation from training session one to six (ts1–6), averaged for all blocks and all participants**.

The course of GDV during training is depicted in Figure 7. From training session to training session GDV was reduced. All differences except for the differences between training sessions 2 + 3, 4 + 5, 4 + 6 and 5 + 6 became significant with *p* < 0.01 (*Post-Hoc*: LSD).

**Figure 8:**
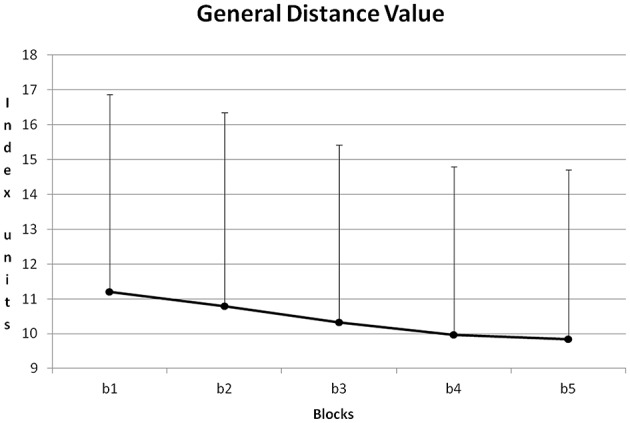
**Course of GDV and its standard deviation during the five blocks of a training session (b1–5), averaged for all training sessions and all participants**.

Figure 8 shows the course of GDV averaged about all training sessions. From block to block GDV was reduced. All differences except for the difference between blocks 4 + 5 became significant with *p* < 0.05 (*Post-Hoc*: LSD).

The learning effect remained stable on retention (three weeks later), no differences between last training and retention became evident, as well in GDV {ANOVA: [*F*_(1, 45)_ = 1.062, *p* = 0.308, $\eta_{\text{p}}^{2}=$ 0.023] as in its subcomponents: grip force [*F*_(1, 45)_ = 2.129, *p* = 0.151, $\eta_{\text{p}}^{2}=$ 0.05], footrest forces [*F*_(1, 45)_ = 0.003, *p* = 0.953, $\eta_{\text{p}}^{2}<$ 0.001], grip pull-out [*F*_(1, 45)_ = 2.549, *p* = 0.117, $\eta_{\text{p}}^{2}=$ 0.05], sliding seat [*F*_(1, 45)_ = 1.450, *p* = 0.235, $\eta_{\text{p}}^{2}=$ 0.03], and force index [*F*_(1, 45)_ = 0.148, *p* = 0.702, $\eta_{\text{p}}^{2}<$ 0.001]}.

### Group differences

**Figure 9:**
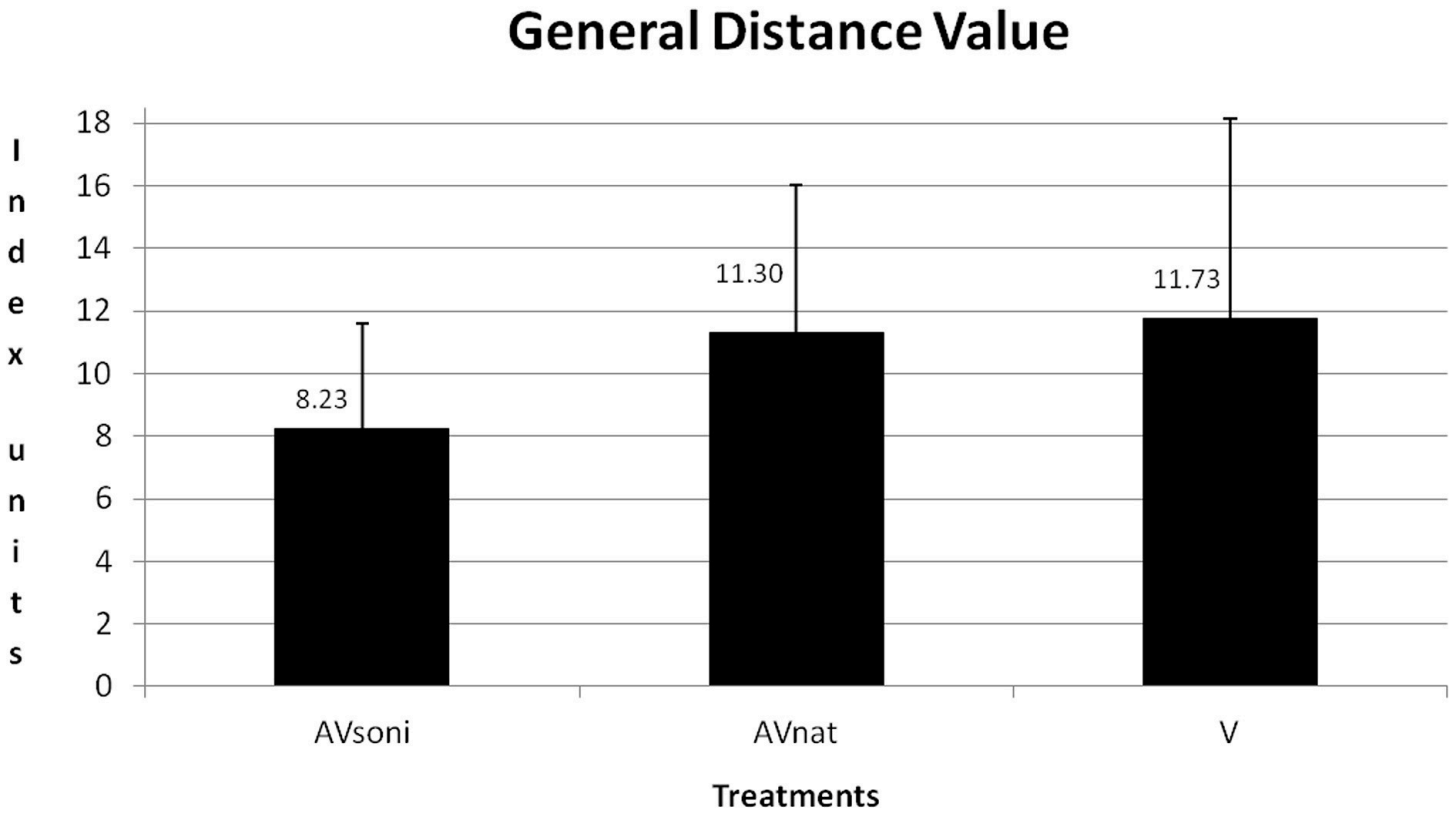
**Group means and standard deviations of GDV for treatment group AV_soni_, AV_nat_, and V averaged for all participants and measurements**.

A scheme about the group differences can be found in Table 1. Main effect “treatment” also became significant [*F*_(2, 45)_ = 3.571, *p* < 0.05, $\eta_{\text{p}}^{2}=$ 0.14]. In Figure 9 the group differences are depicted. In training, AV_soni_ differed from AV_nat_ (*p* = 0.037) as well as from V (*p* = 0.018). AV_nat_ and V didn't differ (*p* = 0.765). (*Post-Hoc*: LSD)

**Table 1:** **Group differences**.

**Component**	**Prob. of error**	**Significant *Post-Hoc* results**
General Distance Value	*p* < 0.05	AV_soni_ – AV_nat_: *p* < 0.05 AV_soni_ – V: *p* < 0.05
Grip pull-out	*p* < 0.001	AV_soni_ – V: *p* < 0.001 AV_nat_ – V: *p* < 0.001
Sliding seat	*p* < 0.001	AV_soni_ – V: *p* < 0.001 AV_soni_ – AV_nat_: *p* < 0.001
Grip force	*p* = 0.096	—
Footrest forces	*p* < 0.001	AV_soni_ – AV_nat_: *p* < 0.001 AV_soni_ – V: *p* < 0.05 V – AV_nat_: *p* < 0.001
Force index	*p* = 0.166	—
Duration of pull-out phase	*p* < 0.001	AV_soni_ – V: *p* < 0.01 AV_nat_ – V: *p* < 0.01
Variability coefficient	*p* = 0.181	—

Probabilities of error (ANOVA, 6^*^5 data points) for main effect treatment and the corresponding Post-Hoc probabilities of error (LSD) in GDV, its subcomponents normalized for pretest (grip pull-out, sliding seat, grip force, footrest forces, and force index) and the additional components normalized for pretest (duration of pull-out phase, variability coefficient). The former treatment group is in each case the one with the lower value.

### Interactions

Interactions with “treatment” didn't become significant {“treatment” × “training session”: [*F*_(10, 225)_ = 0.552, *p* = 0.852, $\eta_{\text{p}}^{2}=0.02$]; “treatment” × “block”: [*F*_(8, 180)_ = 0.658, *p* = 0.728, $\eta_{\text{p}}^{2}=$ 0.03]; “treatment” × “training session” × “block”: [*F*_(40, 900)_ = 0.674, *p* = 0.940, $\eta_{\text{p}}^{2}=$ 0.03], but there was a significant interaction between “training session” and “block” [*F*_(20, 900)_ = 6.722, *p* < 0.001, $\eta_{\text{p}}^{2}=$ 0.13]}.

**Figure 10:**
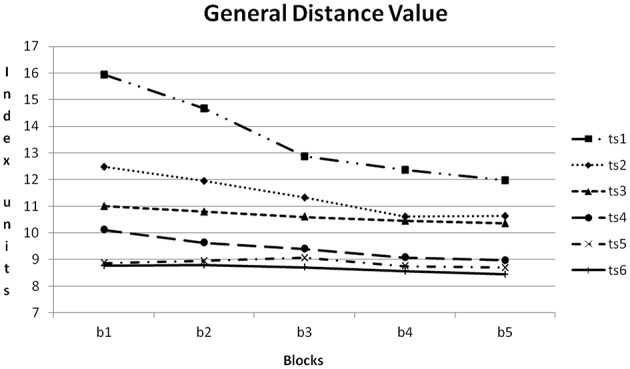
**Course of GDV within the six training sessions (ts1–6) averaged for all participants**. b1–5: blocks 1–5.

In Figure 10 the course of GDV within the six training sessions can be compared. The amount of reduction from training block to training block was reduced from training session to training session. In the first training session reduction was clearly notable, in the sixth training session the course was nearly horizontal.

### Subcomponents

To explore the background of the reported significant main effects, the single components of the GDV (normalized to the group means for pretest) were regarded. For each of the five components, ANOVA revealed significant main effects for “training session” {grip force: [*F*_(5, 225)_ = 21.154, *p* < 0.001, $\eta_{\text{p}}^{2}=$ 0.32]; footrest forces: [*F*_(5, 225)_ = 12.995, *p* < 0.001, $\eta_{\text{p}}^{2}=$ 0.22]; grip pull-out: [*F*_(5, 225)_ = 30.851, *p* < 0.001, $\eta_{\text{p}}^{2}=$ 0.41]; sliding seat: [*F*_(5, 225)_ = 11.088, *p* < 0.001, $\eta_{\text{p}}^{2}=$ 0.20]; force index: [*F*_(5, 225)_ = 48.813, *p* < 0.001, $\eta_{\text{p}}^{2}=$ 0.52]} and for block {grip force: [*F*_(4, 180)_ = 15.749, *p* < 0.001, $\eta_{\text{p}}^{2}=$ 0.26]; footrest forces: [*F*_(4, 180)_ = 8.864, *p* < 0.001, $\eta_{\text{p}}^{2}=$ 0.16]; grip pull-out: [*F*_(4, 180)_ = 29.992, *p* < 0.001, $\eta_{\text{p}}^{2}=$ 0.40]; sliding seat: [*F*_(4, 180)_ = 9.589, *p* < 0.001, $\eta_{\text{p}}^{2}=$ 0.18]; force index: [*F*_(4, 180)_ = 24.629, *p* < 0.001, $\eta_{\text{p}}^{2}=$ 0.35]}, as well as for the interaction “training session” × “block” {grip force: [*F*_(20, 900)_ = 4.682, *p* < 0.001, $\eta_{\text{p}}^{2}=$0.09]; footrest forces: [*F*_(20, 900)_ = 3.519, *p* < 0.001, $\eta_{\text{p}}^{2}=$ 0.07]; grip pull-out: [*F*_(20, 900)_ = 5.801, *p* < 0.001, $\eta_{\text{p}}^{2}=$ 0.11]; sliding seat: [*F*_(20, 900)_ = 1.796, *p* < 0.05, $\eta_{\text{p}}^{2}=$ 0.04]; force index: [*F*_(20, 900)_ = 5.272, *p* < 0.001, $\eta_{\text{p}}^{2}=$ 0.10]}, all with the same tendency as GDV.

Main effect “treatment” however differed between the components: for force index [*F*_(2, 45)_ = 1.866, *p* = 0.166, $\eta_{\text{p}}^{2}=$ 0.08] and grip force [*F*_(2, 45)_ = 2.474, *p* = 0.096, $\eta_{\text{p}}^{2}=$ 0.10] it didn't reach significance, for footrest forces [*F*_(2, 45)_ = 18.380, *p* < 0.001 (Bonferroni adjusted *p*-value), $\eta_{\text{p}}^{2}=$0.45], grip pull-out [*F*_(2, 45)_ = 19.453, *p* < 0.001, $\eta_{\text{p}}^{2}=$ 0.46] and sliding seat [*F*_(2, 45)_ = 23.065, *p* < 0.001 (Bonferroni adjusted *p*-value), $\eta_{\text{p}}^{2}=$ 0.51] it reached level of significance. As we use both “footrest forces” and “sliding seat” to test hypothesis H3, the Bonferroni-adjusted *p*-values are computed for these two components.

**Figure 11:**
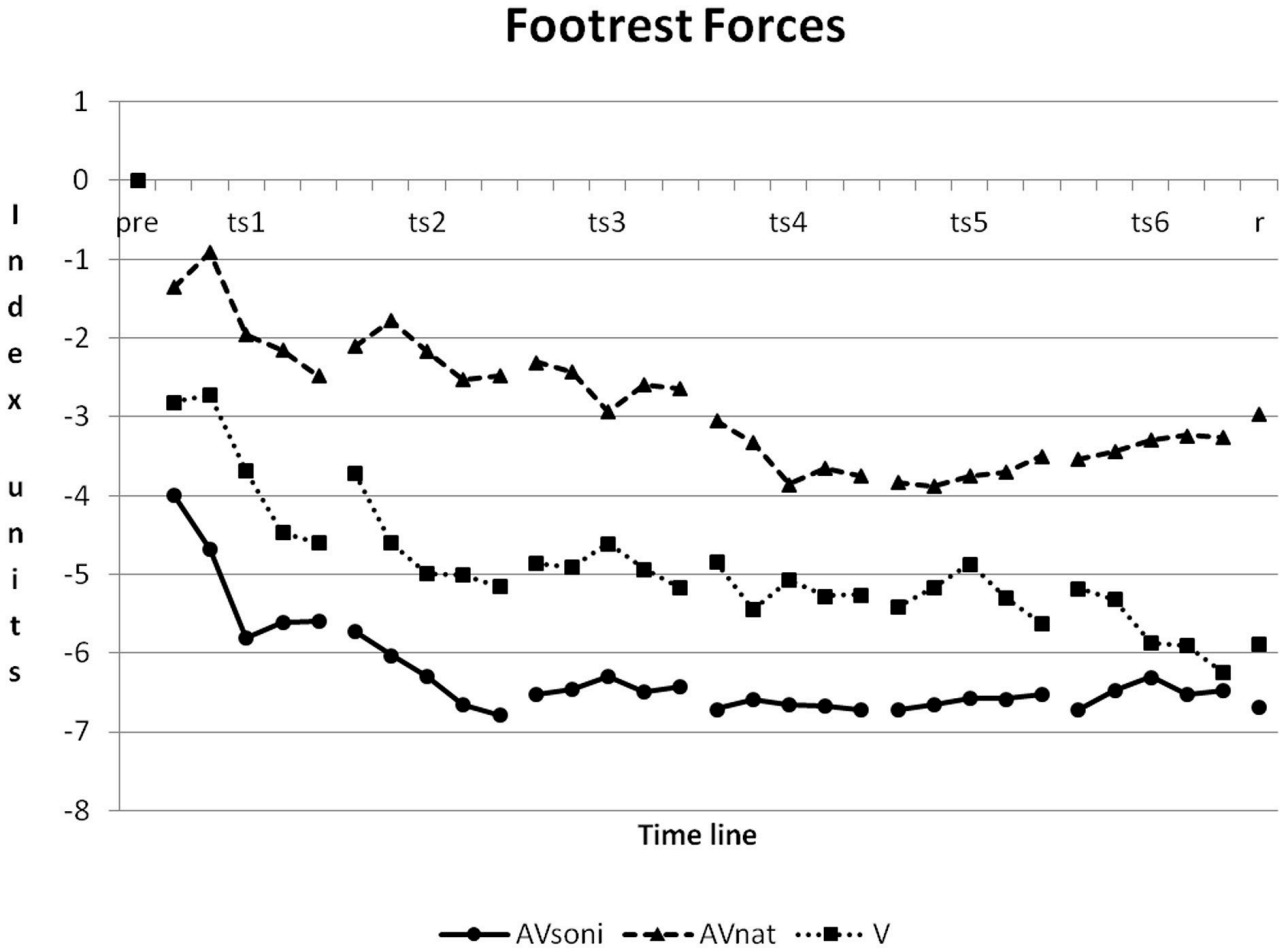
**Development of footrest forces from pretest to retention test for treatment group AV_soni_, AV_nat_, and V (group means of normalized data)**. Pre, pretest; ts, training session; r, retention test.

On footrest forces all three treatment groups differed from each other, AV_soni_ from AV_nat_ (*p* < 0.001), AV_soni_ from V (*p* < 0.05), and AV_nat_ from V (*p* < 0.001) (*Post-Hoc*: LSD). Footrest forces featured the strongest improvement in AV_soni_ and they featured the lowest improvement in AV_*nat*_, as it is depicted in Figure 11.

On grip pull-out V differed from AV_soni_ (*p* < 0.001) as well as from AV_nat_ (*p* < 0.001) (*Post-Hoc*: LSD) with a worse performance for V and on sliding seat AV_soni_ differed from V (*p* < 0.001) as well as from AV_nat_ (*p* < 0.001) (*Post-Hoc*: LSD) with a better performance for AV_soni_.

### Additional data

Additionally two components of the movement that were not integrated in the GDV were regarded: duration of pull-out phase and variability coefficient.

#### Duration of pull-out phase

**Figure 12:**
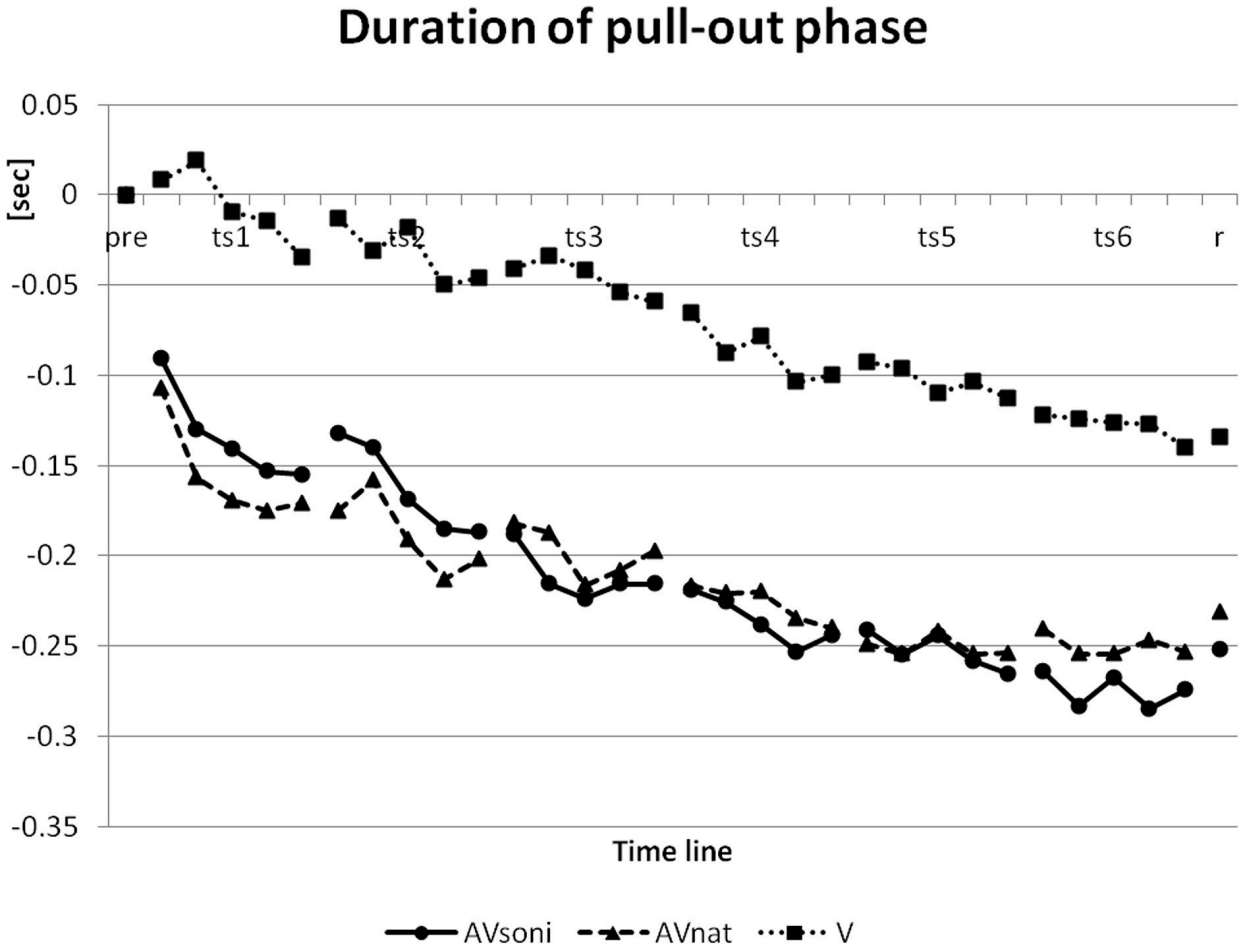
**Development of duration of pull-out phase from pretest to retention test for treatment group AV_*soni*_, AV_*nat*_, and V (group means of normalized data)**. pre, pretest; ts, training session; r, retention test.

In pretest, duration of pull-out phase had a mean of 1.47 s. This data was reduced in the course of the training to a minimum value of 1.25 s in the last block of the last training session and reached a value of 1.26 s in retention test. (The model's pull-out phase and recovery phase had a time ratio of 2:1 with 1 s for pull-out phase). For convenience, a lower value can be considered as better because only three participants ever reached a value of less than 1 s (the model's value). On duration of the pull-out phase (normalized to the group means for pretest) ANOVA revealed a significant main effect “training session”[*F*_(5, 225)_ = 28.387, *p* < 0.001, $\eta_{\text{p}}^{2}=$ 0.39], “block” [*F*_(4, 180)_ = 16.707, *p* < 0.001, $\eta_{\text{p}}^{2}=$ 0.27], and “treatment” [*F*_(2, 45)_ = 6.347, *p* < 0.01, $\eta_{\text{p}}^{2}=$ 0.22] and a significant interaction “training session” × “block” [*F*_(20, 900)_ = 1.867, *p* < 0.05, $\eta_{\text{p}}^{2}=$ 0.04]. V differed from AV_soni_ (*p* < 0.01) as well as from AV_nat_ (*p* < 0.01) (*Post-Hoc*: LSD) with a worse performance for V as it is depicted in Figure 12.

#### Variability coefficient

On variability coefficient (normalized to the group means for pretest) ANOVA revealed a significant main effect “training session”[*F*_(5, 225)_ = 22.842, *p* < 0.001, $\eta_{\text{p}}^{2}=$ 0.34] and “block” [*F*_(4, 180)_ = 8.658, *p* < 0.001, $\eta_{\text{p}}^{2}=$ 0.16]. Main effect “treatment” [*F*_(2, 45)_ = 1.773, *p* = 0.181, $\eta_{\text{p}}^{2}=$ 0.07] and interaction “training session” x “block” [*F*_(20, 900)_ = 1.504, *p* = 0.072, $\eta_{\text{p}}^{2}=$ 0.03] didn't reach significance.

### Retention test

Main effect “treatment” persisted in retention test for GDV {ANOVA: [*F*_(2, 45)_ = 3.707, *p* < 0.05, $\eta_{\text{p}}^{2}=$0.14]} and also for the single components sliding seat [*F*_(2, 45)_ = 19.875, *p* < 0.001, $\eta_{\text{p}}^{2}=$ 0.47], footrest forces [*F*_(2, 45)_ = 19.984, *p* < 0.001, $\eta_{\text{p}}^{2}=$ 0.47], and grip pull-out [*F*_(2, 45)_ = 10.209, *p* < 0.001, $\eta_{\text{p}}^{2}=$ 0.31] but not for the components force index [*F*_(2, 45)_ = 1.390, *p* = 0.259, $\eta_{\text{p}}^{2}=$ 0.06] and grip force [*F*_(2, 45)_ = 0.231, *p* = 0.795, $\eta_{\text{p}}^{2}=$ 0.01].

### Standard deviations

**Figure 13:**
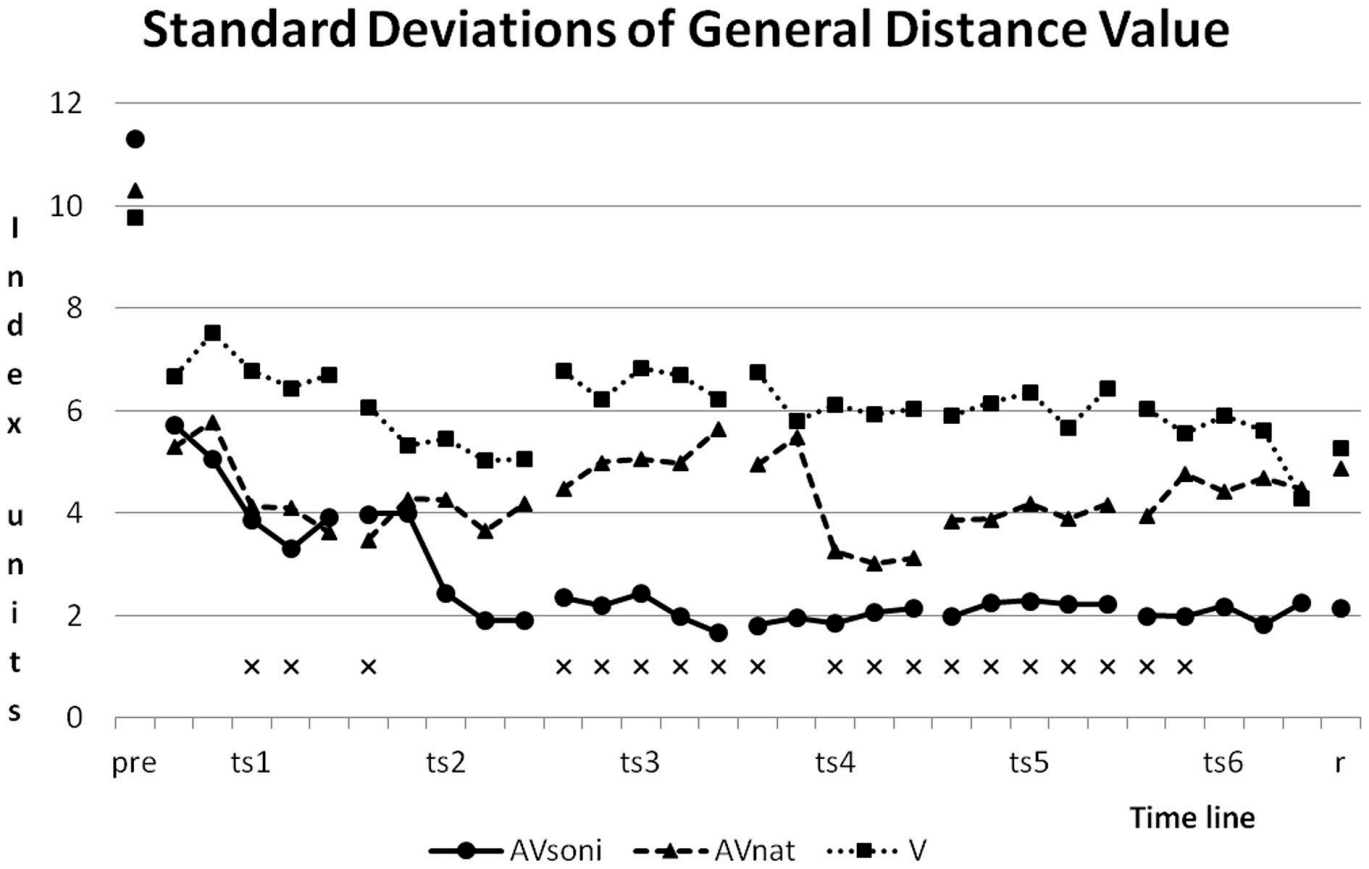
**Development of Standard Deviations of GDV for the three treatment groups**. ^x^mark measuring points with significant results of Levene's Test for Homogeneity of Variances.

As Figure 13 shows, standard deviations developed differently in the three treatment groups. Although group AV_soni_ started with highest value of SD, from training block two in training session two and also in retention test, it had smallest values compared to the other two groups. Group V shows the smallest reduction of SD. Levene's Test for Homogeneity of Variances was significant at 19 and not significant at 11 measuring points.

### Force index and strength ability

**Figure 14:**
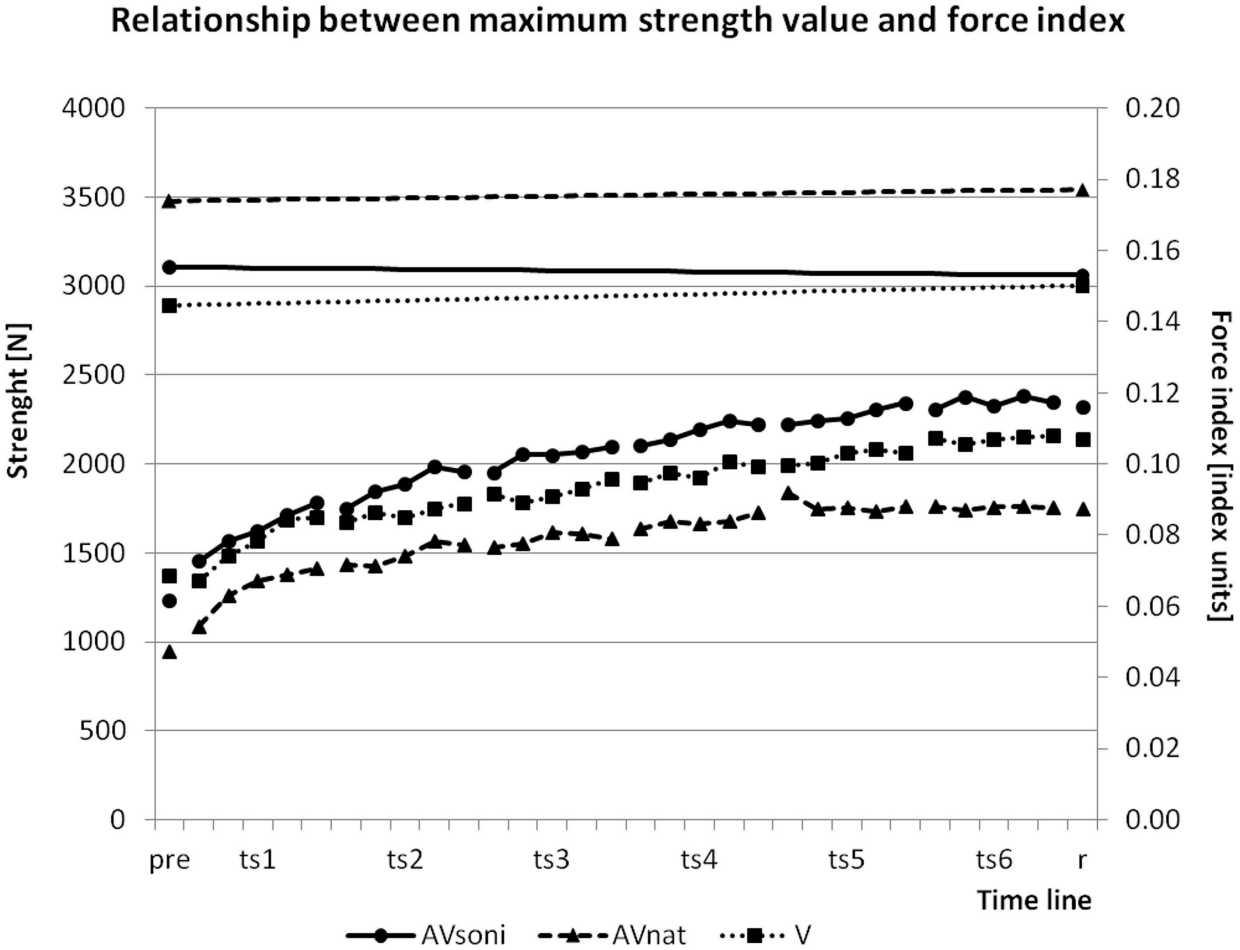
**Changing of group means of maximum strength value (upper part with left ordinate) and development of group means of force index (FI) in the course of the training (lower part with right ordinate) from pretest (pre) via all blocks of all training sessions (ts1-6) to retention test (r)**.

To assure, that the observed growth of the force index during training is not only determined by an improvement of the maximum strength ability, we controlled maximum strength values before and after training. Sums of maximum strength in leg extension and arm flexion before and after training were compared. ANOVA revealed no significant difference between the two measurements [*F*_(1, 45)_ = 0.707, *p* = 0.405, $\eta_{\text{p}}^{2}=$ 0.02]. Figure 14 shows the changing of the maximum strength in relation to the development of FI. While FI was growing in the course of the training, maximum strength remained nearly unchanged.

## Discussion

The present study was conducted to explore the impact of a 4-dimensional movement sonification on the acquisition of the basic technique of indoor-rowing (motor learning). We hypothesized that an extension of visual instruction and feedback with an artificial movement-acoustics would support motor learning. All three experimental groups of male rowing novices observed a video of a rowing professional for instruction and their own movement execution on a projection screen for video feedback, but differed on quantity and quality of additional acoustic movement information. The first group (V) only got visual information. The second group additionally heard the respective natural motion attendant sounds (AV_nat_) and the third group additionally received the movement sonification (AV_soni_). For both audiovisual groups (AV_soni_, AV_nat_) an enhanced learning performance in terms of a sharper learning curve, indicating an increased approximation to the model's technique, was expected compared to the visual group (V).

For the whole sample consisting of male pupils and male students, measurement of initial technical level revealed large differences between participants. We attribute these differences to the wide age range and broad individual differences on coordinative skill level. Despite the heterogeneity of the sample, ANOVA r.m. for GDV revealed significant main effects “training session,” “block” and “treatment”. After 3-week training, a large enhancement in technical level from pretest to last training session was found, indicating for all three training modes a high efficiency on the autonomous acquisition of the rowing technique. Already a remarkable amount of enhancement from pretest to first training session became evident, confirming the novice status of the participants. But besides this, the “jump” of approximation between pretest and first training can be additionally interpreted as an initial effect of the available information on the internal forward dynamic model (Wolpert et al., 2001): Since no feedback was given in the pretest it is plausible, that the instructive information is used for the support of the forward-model. The inverse model should be additionally supported not before feedback was given, that means, not before the middle of the first training block.

Regarding the temporal course, differences between the single training sessions became evident, partly the improvement could be already observed between two consecutive training sessions. In the further study, the learning effect decreases, which might indicate that the realized method was appropriate for the participants. Also within single training sessions an improvement occurred, indicating short-term learning. The amount of the training effect was not constant: Within a single training session the training effect decreased from session to session probably indicating also a short-term learning ceiling effect (see Figure 10 General Distance Value). Between training session five and six almost no improvement became observable any more, probably indicating a general ceiling effect of the chosen autonomous learning setting. Finally the retention measurement confirmed that the achieved learning effects remained stable and were not restricted to the 3-week-training period.

### Subcomponents

The influence of single subcomponents of the movement on the results will be regarded to get additional insights into the development of rowing coordination. Main effects “training” and “block” on GDV can't be attributed to single subcomponents but are reflected in every single subcomponent (grip force, footrest forces, grip pull-out, sliding seat, force index) and also in the additionally regarded movement components, as the duration of the pull-out and the variability coefficient. We interpret this result as an indication for an extensive support of the development of the technique specific coordination via available information. To explore which kind of treatment affects a certain technical feature to what extent, it will be regarded in a first step, how the different treatments are effective in mediating the basic rhythmic structure. Regarding the basic rhythmic structure of a rowing cycle, the expert rower or the model resp., who was performing ergometer rowing with a frequency of about 20 cycles/min resulting in a duration of about 3.0 s for each cycle, realized a basic phase structure of about 1:2, meaning the pull-out phase was about 1 s on average and the recovery phase was about 2 s (pull-out phase: Starting with the local minimum till the local maximum of the pull-out length is reached). The initial phase structure of the total sample showed a nearly 1:1 relation with about 1.47 s on average (initial values: 1.47 s AV_soni_, 1.53 s AV_nat_, 1.41 s V) for pull-out phase, meaning that novices took too much time for the pull-out and might not have been aware of the basic rhythmic structure of the demonstrated rowing motion.

The temporal development of this basic rhythmic movement structure over the training period is shown in Figure 12 (Duration of pull-out phase) based on the normalized samples on the group mean value of the pretest. A clear decrease of pull-out phase duration became evident, resulting in about 1.25 sec on average (final values: 1.19 s AV_soni_, 1.28 s AV_nat_, 1.28 s V) for pull-out phase in the last training block. Furthermore, main effect “treatment” revealed differences between groups with lowest decrease for V compared to both other groups AV_soni_ and AV_nat_. The mean temporal decrease of the pull-out phase duration of all training measures was 0.21 s for AV_soni_, 0.21 s also for AV_nat_ and 0.07 s for V which can be interpreted as a clear auditory or audiovisual benefit on the mediation of the basic movement rhythm compared to a merely visual condition. These findings do confirm hypothesis H2 (Audiovisually treated groups are more precise in rhythmic demands of the indoor rowing technique.).

Furthermore, the findings closely correspond with the ANOVA results concerning the course of the grip pull-out, exemplifying an enhanced approximation of both audiovisual groups to the model compared to the visual group. Natural motion attendant sounds as well as movement sonification in combination with visual movement information seem to provide rhythmic information, that is not available to this extend in the pure visual condition. Taken together, the findings on the duration of the pull-out phase as well as on the course of the grip pull-out clearly support a higher efficiency of an audiovisually based motor learning compared to a merely visual setting, but it cannot explain, on the other hand, the observed group differences of the technique acquisition between AV_soni_ and AV_nat_.

### Main effect “treatment”

When subsequently focusing on the technique acquisition as a whole by regarding the GDV, main effect “treatment” became significant. *Post-hoc* analysis indicated a better learning performance of the AV_soni_ group compared to both other groups (AV_nat_, V), as already described in the “Results”-section. This result pattern is not reflected in the previously regarded technical components “duration of grip pull-out” and “course of grip pull-out,” but in the technical components “sliding seat” and “footrest forces”: For both components the *post-hoc* analysis revealed a superiority for the AV_soni_ group compared to both other groups (AV_*nat*_, V). Whereas the duration and the course of the grip pull-out do not directly refer to the leg motion and the transmission of the force from the footrest to the grip resp., “footrest forces” and “sliding seat” are both features explicitly referring to the kinematic chain “legs-trunk-arm” or to whole body coordination resp., which is the key feature of the rowing technique. The existence of a quite close binding of auditory and motor sequences has just been described by Rauschecker associated with the auditory dorsal stream (premotor-basal-ganglia circuits together with higher auditory centers), which “transforms musical into motor sequence information and vice versa, realizing what are known as forward and inverse models. The basal ganglia and the cerebellum are involved in setting up the sensorimotor associations, translating timing information into spatial codes and back again.” (2014, 1). Hypothesis H3 (Participants treated with complex sonification benefit in terms of a better coordination of the movement resulting in higher technical performance.) is confirmed based on this interpretation of the reported findings (Rauschecker, 2014).

Even though these observation might work as an explanation for parts of the learning effects observed in our study, it has not cleared yet what acoustic or musical features are related to which features of motor execution—which would be indeed a valuable framework for the designing of efficient mapping patterns of movement sonifications. Nevertheless, the findings of Rauschecker (2014) mentioned above can be interpreted to mean that complex musical structures can easily be learned and remembered, carrying a huge amount of sensorimotor information and are usually closely linked to inverse internal models. If a sonification is designed in a consistent and complex mode, as it was realized here in first steps with a mapping of amplitude, frequency and timbre to four certain discrete and continuous movement features, the emerging 4-dimensional movement sonification exhibits quasi-musical features like tempo and rhythm and also some simple melody. This complex and quasi-musical character of the sonification might be an explanation for the observed effects surpassing the effects of rhythmic adjustments.

This kind of complex information is visually obviously not available with comparable precision and it is also not included in the auditory information that natural motion attendant sounds provide. Obviously, a larger informational content is decoded from complex movement sonification compared to natural or more reduced forms of movement acoustics. This assumption is supported by two additional findings: (1) Group differences are also preserved in retention test 3 weeks after last training session, indicating an outlasting learning advantage for participants treated with additional sonification. (2) Interestingly, for the AV_soni_ group the approximation to the model's technique during training seems to be accompanied by a reduction of interindividual heterogeneity, which seems to be given for the AV_soni_ group as illustrated in Figure 13 (Standard deviations of General Distance Value).

An alternative explanation could have been that participants did not improve their rowing technique but instead their strength capacity and thus were able to approximate increasingly to the model's technique. But because maximum strength values did not change from pre- to posttest, the increase of used force (FI) can't be attributed to an increase of the participants' maximum strength capacities. A more plausible explanation would be that an improvement of a force demanding movement technique can be understood as a better utilization of already existing strength capacities by a smoother and more economical coordination when executing the technique, as illustrated in Figure 14 (Relationship between maximum force value and force index).

To control if the expected differences between the merely visually treated group and the audiovisual sonification group can be explained by the addition of a further sense alone or by the specific shape of the sonification, we created an audiovisual control group with another 16 participants, who heard the natural motion attendant sounds besides seeing the video as instruction and feedback. This group also showed a worse learning performance compared to the audiovisually treated sonification group in terms of a larger technical distance to the model over the whole course of the study. This finding is somewhat surprising at a first sight, because it became evident (see section Non-Musical Acoustic Information on Motor Behavior) that also natural movement sounds carry a lot of information which can be decoded by the auditory system easily and used to enable or modulate movement perception as well as motor control. So how to explain the measured differences? Given that natural motion attendant sounds are integrated presumably not worse with visual information in humans than sonification is, sonification apparently provides more information about the movement than natural motion attendant sounds do. The latter consisted of the sound of the rowing ergometer flywheel and the sliding seat. In comparison to sonification, these sounds also contain information about the grip force (flywheel sound), the movement of the sliding seat and the temporal relation between both. But there was no acoustic information about the footrest force nor about the grip pull-out for this group, which might explain the reduced amount of information extractable from the natural motion attendant sounds. Hypothesis H1 (Participants of both groups treated with convergent audiovisual information show better learning results in terms of a steeper learning curve/a faster approximation to the model technique.) cannot be confirmed based on these findings.

### Neurophysiological framework

In summary, movement sonification in combination with video provides information that is neither available visually (video alone) nor with video combined with natural motion attendant sounds. For the alignment of one's own action to a visual, auditory or audiovisual model action, the mirror neuron system is important. In our study, in that the rowing ergometer is tuned into a sound instrument, participants of the sonification group were able to perceive rowing action and sonification for altogether 45 min synchronously. As the selected movement features were coupled to certain acoustic features in a fixed mode, it is plausible that movement specific audio-motor co-activation patterns emerge in the CNS: Such audio-motor co-activation networks can emerge even within about first 20 min of practice, as shown by Bangert and Altenmüller (2003) on music novices. The benefit of movement sonification on motor perception, re-enactment (Effenberg, 2005; Young et al., 2013) and synchronization (Schaffert et al., 2011) was established before. A motor learning study of Sigrist et al. (2015)—enhancing the horizontal angle of the rowing oar during the recovery phase in indoor rowing—failed to generate a long-lasting learning effect with error-feedback. In contrast to the use of sonification as error-feedback in rowing, usually requiring conscious cognitive processing, here a complex movement sonification is created to enhance and accelerate the emergence of adequate internal representations of the new movement technique. This should be achieved by a movement sonification which is comprehensively integrable with perceptual streams of other modalities as visual and kinesthetic as the most important ones. This way, the movement sonification should have supported the emergence of the forward model in a first step when used in the instructive mode. But though the rowing model as well as the participants' own rowing motion were sonified in an equivalent manner (all parameter sets were normalized before post-processing), also the inverse model should have been supported when sonification was used as additional real-time feedback during movement execution. These mechanisms might have been also responsible for the effects of real-time movement sonification we observed recently for the acquisition of character handwriting on children (Effenberg et al., 2015).

Even though multisensory integration efficiency of the generated audiovisual stimuli was not investigated in this behavioral study, former fMRI-research of our workgroup already confirmed the integration efficiency of such type of intermodal convergently shaped movement sonification based on dynamic (Scheef et al., 2009) and kinematic (Schmitz et al., 2013) movement parameters. Here, evidence was presented that learning of a complex gross motor movement can be improved by this type of movement information. Explicit knowledge about the mapping doesn't seem to be required for using this information on motor learning. It was shown that the effects were surpassing effects of rhythmic adaptation and that they were long-lasting. Additionally, there are some indications that the efficiency might be independent from directing conscious attention to it, which would be in line with the idea that at least bottom-up proportions of multisensory integration are dependent primarily on temporal and spatial stimulus convergence combined with structural analogy demands (content-related congruity) on perceived stimuli (Calvert et al., 2001). Intermodal convergent sonification seems to have an implicit informational effect, emerging from integration with visual and kinesthetic movement information, when matching to the observed movement just as natural motion attendant sounds do match.

Since movement sonification was configured with high-degree of convergence to visual percept, it could be suggested that beside audiomotor mechanisms, audio-visuo-motor mechanisms were involved in copying the model's rowing technique. Kaplan and Iacoboni (2007) found a region in ventral premotor cortex that shows a specific response on the conjunction of visual and auditory action-related stimuli and thus might produce a modality-independent representation of the action. Since the ventral premotor cortex is involved in action planning, the findings indicate that the investigated region contributes to the representation of actions, independent of agency and sensory modality. Hence, although there is no direct linkage between the movement and the sonification, the visual event as mediating link might facilitate the establishment of such a linkage in the brain. This could be an explanation for the fact that a learning advantage of the sonification group occurred already in the first training session, that means after 60 s of exposure to sonification (30 s instruction and 30 s feedback).

### Conclusion

Finally, it should be emphasized that the introduced method of intermodal convergent real-time movement sonification should be also adaptable to motor rehabilitation, such as for stroke rehabilitation (hemiparesis) or gait rehabilitation after endoprothesis. If effectiveness of the method is at least partially based on direct multimodal integration as described here, it might work below the level of consciousness. For this reason, it should be also effective especially on patients with certain sensorimotor restrictions as in Parkinson's disease (Thaut et al., 1996) e.g., recently theoretically underpinned by Murgia et al. (2015) with strong references to “rhythmic auditory stimulation” established by Thaut et al. (1997)—and even beyond rhythmic adjustments. Additional empirical work of Young et al. (2014) indicates the efficiency of auditory step models on the gait of Parkinson patients. But multidimensional kinds of real-time movement sonification containing continuously mapped parameters even exceed rhythmic adjustments by addressing kinematic chains or whole body coordination, as discussed in section “Main effect Treatment.” Besides the findings presented here, there is further initial evidence from our workgroup on motor rehabilitation of the upper limb on hemiparesis patients (Schmitz et al., 2014). In the future, it should be possible to further improve the efficiency of established methods by adding real-time movement sonification as described in this paper. What has not been proven yet is the effectiveness of auditory movement information alone—how movement sonification might support motor learning as a substitution of visual information. Further research should be directed to such questions as well as to different fields of motor rehabilitation, currently only sparsely supported by initial indications of intermodal information processing.

## Author contributions

AE created the topic, developed the method and was concerned with the data collection and the statistical analysis of the data. He did write broad parts of the paper and was the responsible author together with UF. UF was supporting when creating the topic, she was collecting the data and was computing the statistical analysis of the data together with AE and GS. She did also write broad parts of the paper and was the responsible co-author together with AE. Both authors (AE and UF) contributed equally to this work. GS was supporting the statistical analysis of the data. He gave significant input to some passages of the text. BK was developing parts of the sonification-system used in the study as well as the dtw-algorithm. HM was the supervisor of the project. He was supporting the development of the whole method as well as the organization of the data collection.

### Conflict of interest statement

The authors declare that the research was conducted in the absence of any commercial or financial relationships that could be construed as a potential conflict of interest.

## Footnotes

^1^Standard vision and hearing test: HTTS, Version 2.10, 00115.04711.

^2^Behringer, ECM 8000.

^3^beyer dynamic DT 100.

^4^FES, Ruderergometer Version 2.43.

^5^National Instruments, LabVIEW 7.1.

^6^MLmini, Universität Bonn, Institut für Informatik, AG Prof. Weber.

^7^Junior world champion 2005, 4+; world champion 2011 8+; Olympic Games champion 2012 8+.
